# Organic Bioelectronics

**DOI:** 10.1002/advs.202404458

**Published:** 2024-06-28

**Authors:** Mohammad Reza Abidian

**Affiliations:** ^1^ Department of Biomedical Engineering University of Houston 3517 Cullen Blvd. Houston TX 77204 USA

This special issue dedicates to the emerging field of organic bioelectronics that explore the fundamentals and the applications of organic electronics materials in biology and medicine. Bridging the interface between biology and electronics necessitates advances in materials, enabling better performing devices or entirely new device concepts. Organic bioelectronics include the design and development of organic electronic materials that operate as translators between biological signals and human‐made electronics. The key advantage of organic semiconductors in bioelectronics is that they can be designed at the molecular level to mimic mechanical properties of biological tissue, modulate biological responses, and transport both ionic and electronic carriers.^[^ ^1^ ^]^ Over the past few years, organic electronic materials have demonstrated excellent optoelectronic properties that have led to the development of light emitting diodes, thin field‐effect transistors, and photovoltaics for consumer electronics and energy‐related applications. The mixed conduction of electronic and ionic charge carriers in organic semiconductors has paved the way for applications in bioelectronics including biosensors, neural interfaces, drug delivery devices, and tissue engineering constructs.^[^ ^2^ ^]^ This special issue includes review papers and research articles that highlight the fundamentals and the applications of organic electronics to bioelectronics, while at the same time bringing to the foreground some important implications for the molecular level design of materials and devices.

This special issue contains 21 articles (17 Research Articles and 4 Reviews), including, organic electrochemical transistors for biomarker detections, organic bioelectronic sensors, and organic bioelectronic neural interfaces. Prof. Pool‐Warren and co‐workers (article number 202306275) conduct a comprehensive review on organic bioelectronic neural interfaces and focus on the potential benefits of organic electrode coatings and the challenges that must be overcome to implement them in current implantable devices. Prof. Oh and co‐workers (article number 202306191) perform a comprehensive review on organic mixed ionic–electronic conductors for bioelectronic sensors. They present an in‐depth review of recent research achievements in organic bioelectronic applications using organic mixed ionic‐electronic conductors that are categorized based on physical and chemical stimuli as well as neuromorphic devices and circuit applications. Prof. Sonar (article number 202305611) in a review shed light on advanced neuromorphic applications enabled by synaptic ion‐gating vertical transistors. Prof. Yan and co‐workers (article number 202305347) further review organic electrochemical transistors for biomarker detections, with a focus on the recent advances and representative applications organic electrochemical transistors in wearable and implantable devices. Prof. Malliaras and co‐workers (article number 202301176) report a high‐density conformable body surface potential mapping with conducting polymer‐eutectogel electrode arrays for ECG imaging. In another research article, Prof. Malliaras and co‐workers (article number 202306424) fabricate a light‐based 3D multimaterial printing of microstructured conducting and dry adhesive electrodes for bioelectronic applications. Prof. Rogers and co‐workers (article number 202301232) demonstrate implantable, bioresorbable radio frequency resonant circuits for magnetic resonance imaging. Their results suggest that this technology has a broad potential for postsurgical monitoring of recovery processes. Prof. Owens and co‐workers (article number 202304301) construct microelectrode arrays to measure blocking of voltage‐gated calcium‐ion channels on supported lipid bilayers that are derived from primary neurons. Prof. Lessard and co‐workers (article number 202305515) report an axial phenoxylation of aluminum phthalocyanines for improved cannabinoid sensitivity in organic thin‐film transistors. Prof. Rivnay and co‐ workers (article number 202305562) present a method for decoupling of poly(3,4‐ ethylenedioxythiophene)‐collagen composite characteristics on cell stemness. Prof. Santoro and co‐workers (article number 202305860) develop a concealing organic neuromorphic device with neuronal‐inspired supported lipid bilayers that can potentially be further exploited for assembling of hybrid neuronal networks and potentially for in vivo integration within living neuronal tissues. In a research article, Prof. Anthopolous and co‐workers (article number 202306038) fabricate a label‐free ultrasensitive transistor biosensors for metabolites detection in human saliva and show the specificity of the biosensors against various interfering species, including other metabolites, and proteins found in saliva, further showcasing its capabilities. Prof. Asplund and co‐workers (article number 202306244) report a bioelectronic direct current stimulation at the transition between reversible and irreversible charge transfer using conducting polymer poly(3,4‐ethylenedioxythiophene) (PEDOT). Their results suggest that although electrode materials generate reactive oxygen species, the onset can be delayed by increasing of electrode's capacitance via PEDOT coating. Prof. Inal and co‐workers (article number 202306716) demonstrate a novel biofunctionalization method for high stability and longevity of electronic biosensors. Their proposed method represents a broadly applicable biofunctionalization technique for enhancing cost effectiveness, sustainability, and longevity of electronic biosensors, all without compromising sensitivity. Prof. Herland and co‐workers (article number 202307042) report a cleanroom‐free direct laser micropatterning method for organic electrochemical transistors in logic circuits and glucose biosensors. Prof. Richter‐Dahlfors and co‐workers (article number 202307322) desaturate that high‐resolution large‐area image analysis deciphers distribution of salmonella cells and ECM components in biofilms on charged PEDOT:PSS surfaces. This research provides yet a link between conducting polymers and bacterial metabolism and shows for the first time a specific effect of electrochemical addressing on bacterial ECM formations. Prof. Khodagholy and co‐workers (article number 202308014) reveal formation of anisotropic conducting interlayers for high‐resolution epidermal electromyography using mixed‐conducting particulate composite. Prof. Torsi and co‐workers (article number 202308141) present a method for analysis of clinical samples of pancreatic cyst's lesions with a multianalyte SiMoT array benchmarked against ultrasensitive chemiluminescent. In a research article, Prof. Cui and co‐workers (article number 202308212) showcase that PEDOT‐carbon nanotube composite microelectrode arrays reveal new insights into the Clock gene's role in dopamine dynamics in the context of circadian rhythm regulation and chronically reliable performance and dual measurement capability. Prof. van de Burgt and co‐workers (article number 202308261) fabricate organic neuromorphic micro‐networks and implement software‐based neural network and actual measurements that predict blood glucose levels. Prof. Nielson and co‐workers (article number 202308281) construct single‐component electroactive polymer architectures for non‐enzymatic glucose detection with high sensitivity to glucose in the range of 10 µM–10 mM.

To close, I appreciate the kind support from the editorial team of Advanced Science, Dr. Marco Squillaci and Dr. Flora Kiss. In addition, I am very grateful to all the authors, who contributed in this exciting special issue of Organic Bioelectronics.
